# Retraction: Specific inhibition of tumor cells by oncogenic *EGFR* specific silencing by RNA interference

**DOI:** 10.1371/journal.pone.0321802

**Published:** 2025-04-01

**Authors:** The PLOS One Editors

After this article [1] and subsequent Expression of Concern [2] were published, additional concerns were raised about Figures S4 and S14.

## Specifically:

In Figure S4, the following concerns were noted when levels are adjusted to visualize the background:

- The pEGFR panel (PC-3 cells) appears similar to the pAKT panel (PC-3 cells) when flipped vertically and with altered aspect ratio.
- Lanes 2 and 3 in the AKT panel (PC-3 cells) appear similar to lanes 2 and 3 in the EGFR panel (PC-9 cells).
- The TUBULIN panel (PC-3 cells) appears similar to the AKT panel (PC-9 cells).
- There appears to be a vertical discontinuity between lanes 1 and 2 in the ERK1/2 panel (PC-9 cells).

In Figure S14, the following panels appear to partially overlap:

- In the Intestine results, the siControl and siEgfr panels.
- In the Spleen results, the Delivery vehicle and siEgfr panels.

The corresponding author stated that the original images underlying the figures of concern are not available. In the absence of these data, the concerns remain unresolved.

In light of the unresolved concerns noted above and in [2] which call into question the reliability of the reported results and conclusions, the *PLOS One* Editors retract this article.

MT, TO, and HH did not agree with the retraction and apologize for the issues with the published article. TC either did not respond directly or could not be reached.
